# Describing and evaluating healthcare priority setting practices at the county level in Kenya

**DOI:** 10.1002/hpm.2527

**Published:** 2018-04-15

**Authors:** Dennis Waithaka, Benjamin Tsofa, Evelyn Kabia, Edwine Barasa

**Affiliations:** ^1^ Health Systems and Research Ethics Department KEMRI Wellcome Trust Research Programme Kilifi Kenya; ^2^ Health Economics Research Unit KEMRI Wellcome Trust Research Programme Nairobi Kenya; ^3^ Nuffield Department of Medicine University of Oxford Oxford UK

**Keywords:** county, Kenya, planning and budgeting, priority setting

## Abstract

**Background:**

Healthcare priority setting research has focused at the macro (national) and micro (patient level), while there is a dearth of literature on meso‐level (subnational/regional) priority setting practices. In this study, we aimed to describe and evaluate healthcare priority setting practices at the county level in Kenya.

**Methods:**

We used a qualitative case study approach to examine the planning and budgeting processes in 2 counties in Kenya. We collected the data through in‐depth interviews of senior managers, middle‐level managers, frontline managers, and health partners (n = 23) and document reviews. We analyzed the data using a framework approach.

**Findings:**

The planning and budgeting processes in both counties were characterized by misalignment and the dominance of informal considerations in decision making. When evaluated against consequential conditions, efficiency and equity considerations were not incorporated in the planning and budgeting processes. Stakeholders were more satisfied and understood the planning process compared with the budgeting process. There was a lack of shifting of priorities and unsatisfactory implementation of decisions. Against procedural conditions, the planning process was more inclusive and transparent and stakeholders were more empowered compared with the budgeting process. There was ineffective use of data, lack of provisions for appeal and revisions, and limited mechanisms for incorporating community values in the planning and budgeting.

**Conclusion:**

County governments can improve the planning and budgeting processes by aligning them, implementing a systematic priority setting process with explicit resource allocation criteria, and adhering to both consequential and procedural aspects of an ideal priority setting process.

## 1 INTRODUCTION

Priority setting occurs at all levels of the health system and is one of the most important health policy questions in recent years.^1^, ^2^ Priority setting has been recognized as a key determinant of success in healthcare delivery.^3^, ^4^, ^5^ However, health sector priority setting research has focused on macro and micro levels, while neglecting meso‐level priority setting practices.^4^, ^6^, ^7^ Specifically, in low‐ and middle‐income countries (LMICs), there is limited research on priority setting and resource allocation practices at the meso‐level in regional and/or district health systems.^4^, ^8^ This is perhaps surprising given that decentralization has formed a key part of health sector reforms in LMICs.^9^ Decentralized health systems form the focal point of most LMIC health systems and control significant health sector resources. Examples of priority setting activities in the health sector include strategic planning processes, budgeting processes, decision making about development of benefit packages, medicines selection, and distribution and allocation of human resources for health.

This paper examines the planning and budgeting processes at the county level in Kenya as the tracer priority setting activity. In 2013, Kenya transitioned from a centralized to a decentralized system of governance. Under this new governance arrangement, there is a central government and 47 county governments.^10^ Within the health sector, the national Ministry of Health (MOH) retained policy and regulatory roles, while the service delivery role has been transferred to county governments.^11^ County governments own and manage county hospitals that provide secondary care, as well as health centers and dispensaries that provide primary healthcare services. County governments also deliver preventive healthcare services. County Departments of Health (CDOH) coordinate and implement health sector activities in county governments. In the financial year 2016 to 2017, the national government allocated 60 billion Kenya shillings (US $600 million) to the national MOH, while the 47 county governments collectively allocated 90 billion Kenya shillings (US $900 million) to the CDOH.^12^ County Departments of Health, therefore, not only play a critical role in the delivery of healthcare services but also control the greatest proportion of healthcare resources in the Kenyan health system. It is therefore critical that the resources controlled by the CDOH are optimally used to achieve desired healthcare outcomes. Understanding how the CDOH set their priorities and identifying policy recommendations for improving the priority setting practices of the CDOH is therefore a key research question. However, there is a dearth of literature on meso‐level priority setting practices in general^13^ and, in Kenya, no study has evaluated the priority setting practices of the CDOH. There is therefore a need and a knowledge gap in evaluating CDOH priority setting practices. This study sought to contribute to the knowledge on evaluating meso‐level healthcare priority setting processes in general, and in Kenya specifically by describing and evaluating the planning and budgeting processes, as key priority setting processes, in 2 case study counties.

## 2 METHODS

### 2.1 Study design

We used a qualitative case study approach and collected data through in‐depth interviews and document reviews. A case study is “a distinctive empirical inquiry that investigates a contemporary phenomenon in depth and within its real‐life context, especially when the boundaries between phenomenon and context are not clearly evident.”^14^ Further, case study design is fitting for the evaluation of complex situations whereby the output is subject to a range of possible explanations from those involved.^15^ Healthcare priority setting is a complex and contextual social process^16^; therefore, a case study approach offered the opportunity to capture the perspectives and experiences of the stakeholders in their “natural setting.” We examined the CDOH planning and budgeting processes since it is the main health sector priority setting activity at the county level in Kenya.

### 2.2 Study setting

Kenya is a lower‐middle income country with a devolved system of governance organized around 2 major administrative levels: national and county.^10^ Within the health sector, the national government has policy and regulatory roles, while the 47 county governments have healthcare service provision roles.^11^ The provision of healthcare services in the public sector is organized into 4 tiers: tier 1, community health units; tier 2, primary care (dispensaries and health centers); tier 3, secondary care (county hospitals); and tier 4, tertiary care (national referral hospitals).^17^

We conducted the study in 2 counties in coastal Kenya. We purposefully selected the 2 counties based on the following criteria: (1) researcher convenience and access to the county offices, (2) anecdotal evidence that one county had better governance practices than the other, and (3) working relationship with our research institution. This last criterion was important because of the political aspects and sensitivity of the planning and budgeting processes. It also entailed asking and reviewing documents such as plans and budgets. Hence, this would likely encounter different forms of resistance. By identifying counties with prior relationship, it minimized trust concerns that led to a rich source of information. Table 1 presents the characteristics of county A while Table 2 presents the characteristics of county B.

**Table 1:** Key demographic and health indicators in county A

Indicator	County A 2012	County A 2015	Kenya 2015
Population			
Total	1 218 297	1 296 510	45 108 414
Male	587 915	625 658	22 422 667
Female	630 382	670 852	22 685 747
Under 5	1 976 364	210 035	6 936 691
Under 1	42 640	45 378	1 425 787
Health personnel (public)			
Nurses (per 100 000 people)	35	30	55
Doctors (per 100 000 people)	10	4	10
Clinical officers (per 100 000 people)	13	17	21
Health facilities			
Public	93	107	4929
Non‐governmental	6	9	347
Faith based	13	14	1081
Private	110	127	3797
Health financing			
Total government health spending (per capita, Kenya shillings)	808	1433	1585
National Hospital Insurance Fund coverage county (% of county population)	19.0	24.0	26.7

Source: https://www.healthpolicyproject.com/pubs/291/County A%20County-FINAL.pdf

**Table 2:** Key demographic and health indicators in county B

Indicator	County B 2012	County B 2015	Kenya 2015
Population			
Total	713 512	759 318	45 108 414
Male	346 910	369 181	22 422 667
Female	366 602	390 137	22 685 747
Under 5	115 589	123 010	6 936 691
Under 1	24 973	26 576	1 425 787
Health personnel (public)			
Nurses (per 100 000 people)	36	35	55
Doctors (per 100 000 people)	3	4	10
Clinical officers (per 100 000 people)	15	17	21
Health facilities			
Public	62	74	4929
Non‐governmental	2	3	347
Faith based	4	6	1081
Private	24	32	3797
Health financing			
Total government health spending (per capita, Kenya shillings)	894	1561	1585
National Hospital Insurance Fund coverage county (% of county population)	22	28.5	26.7

Source: http://www.healthpolicyproject.com/pubs/291/County B%20County-FINAL.pdf

### 2.3 Data collection procedures

Data collection was done by D.W. We collected data through a combination of in‐depth interviews with senior managers, middle‐level managers, frontline managers, and health partners and review of relevant documents including the county plans and budgets. We purposively selected participants targeting individuals with in‐depth knowledge of the planning and budgeting processes and those who took part in the process within the counties. The final number of participants was dependent on conceptual saturation. The interview guide was piloted on managers in county A and refined. A total of 23 in‐depth interviews were conducted within their work environment lasting approximately 60 minutes. None of the interviewees declined to participate. Table 3 outlines the distribution of participants in the different management levels.

**Table 3:** Distribution of study participants

Management Levels	Number of Participants in County A	Number of Participants in County B
Senior managers	4	5
Middle‐level managers	5	5
Frontline manager	1	2
Health sector partners	1	NA
Study total	23	

### 2.4 Conceptual framework

**Figure 1:**
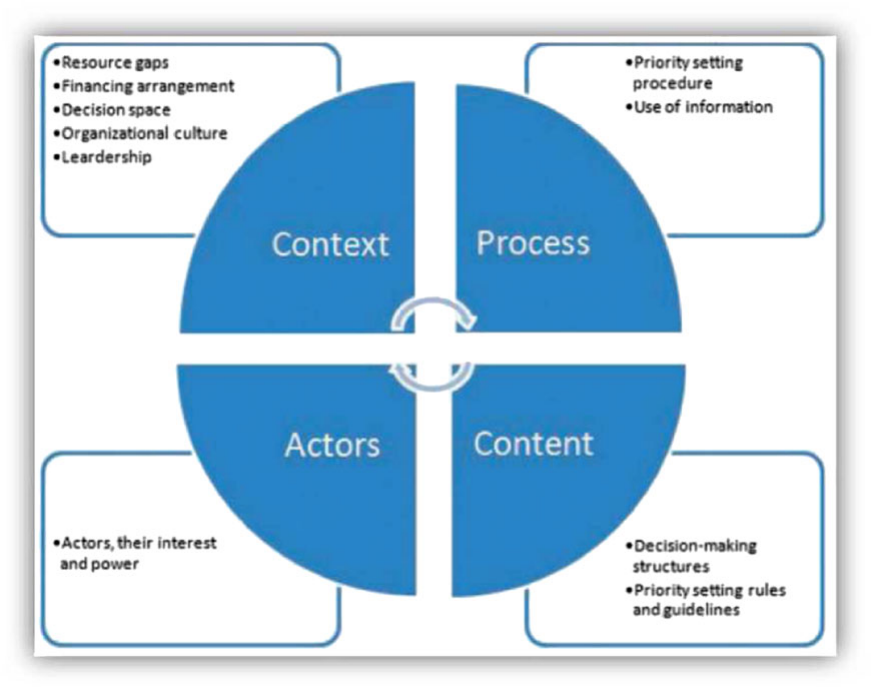
Descriptive framework for priority setting practice [Colour figure can be viewed at http://wileyonlinelibrary.com]

We adopted 2 frameworks to guide the description and evaluation of the planning and budgeting processes at the county level. To describe the planning and budgeting processes, we adapted the priority setting descriptive framework developed by Barasa et al,^8^ which considers priority setting practices to include context, content, process, and actors (Figure 1). The framework examines priority setting in 4 domains as follows. The *context* of priority setting refers to the circumstances that surround the priority setting activity. This includes factors such as resource gaps, financial arrangement, decision space, organizational culture, and leadership. The *content* entails the decision‐making structures and the rules and guidelines that govern the process. The *process* includes the priority setting procedure. *Actors* refer to the various stakeholders, their interests, and power dynamics in the priority setting activity.

**Figure 2:**
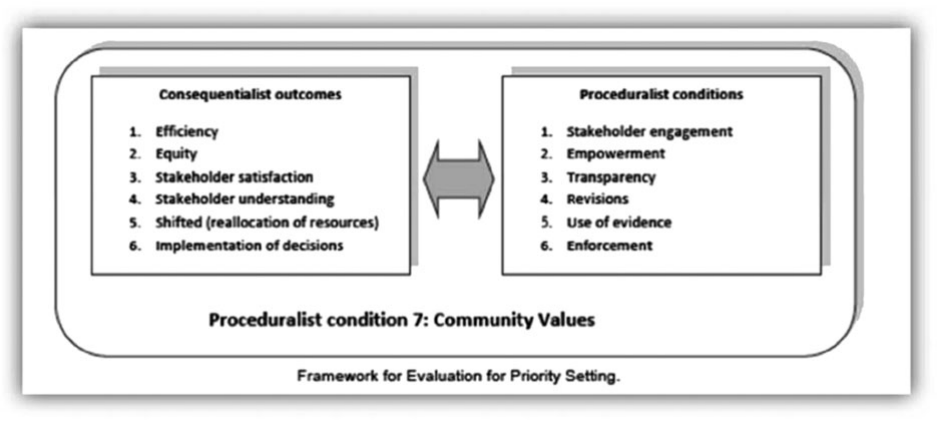
Evaluative framework for priority setting practice

To evaluate the planning and budgeting processes, we used the priority setting evaluation framework developed by Barasa et al^5^ (Figure 2). This framework proposes that an ideal priority setting process should adhere to both consequential and procedural conditions. Consequential conditions refer to the desired outcomes of a priority setting process. These include efficiency, equity, stakeholder satisfaction, stakeholder understanding, shifted (reallocation of) resources, and implementation of decisions.

Procedural conditions refer to aspects of priority setting processes that incorporate deliberative democratic principles. These include stakeholder engagement, stakeholder empowerment, use of information, revisions, transparency, and enforcement.

### 2.5 Data management and analysis

We transcribed the recorded interviews and stored them in password‐protected computers. We safely stored all the field notes, documents for review, and audio recorder under lock and key at all times to ensure confidentiality. Data cleaning was done followed by manual coding by all the authors. We then analyzed the data using a framework approach. The process entailed identifying connections between the data collected and predetermined thematic framework by sifting, sorting, coding, and charting collected data.^18^ This approach was adopted to provide output that will inform policy decisions.

### 2.6 Ethical considerations

This study obtained ethics review and approval from the KEMRI Scientific and Ethics Review Committee (SSC: 2205).

## 3 FINDINGS

### 3.1 Description of the planning and budgeting processes

#### 3.1.1 County department of health decision‐making structure

**Figure 3:**
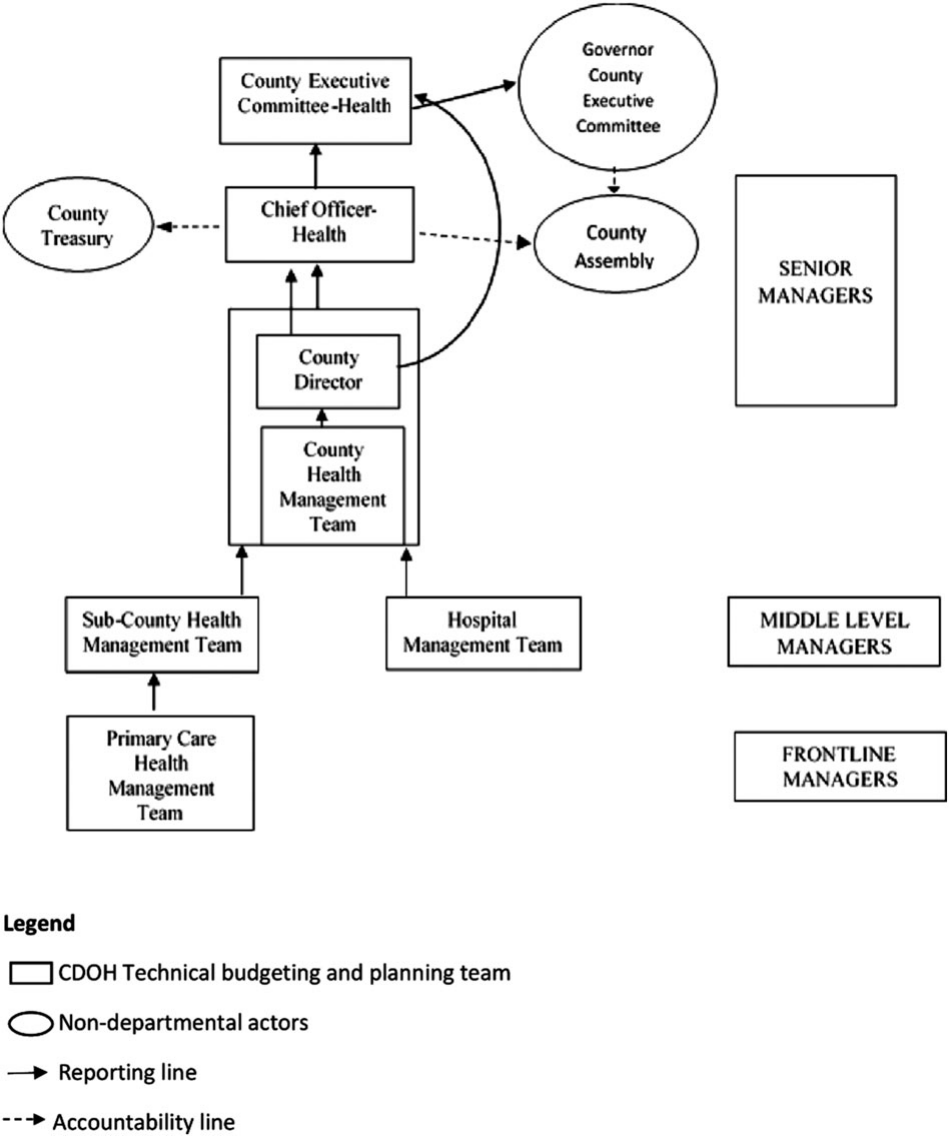
County Departments of Health (CDOH) decision‐making structure

The counties did not have an official organogram. However, observations and discussions with the healthcare managers revealed the existence of 6 technical management levels in the CDOH (Figure 3). At the lowest level was the primary healthcare facility management (PHC‐FMT). The PHC‐FMT is composed of the nursing officer‐in‐charge and the clinical officer‐in‐charge of dispensaries and health centers respectively. The PHC‐FMT was answerable to the subcounty health management team (SCHMT). The SCHMT is composed of all program officers and departmental heads within the subcounty, with the subcounty medical officer of health as the chair. The SCHMT was answerable to the county health management team (CHMT). There was also the hospital management team that is composed of the hospital departmental heads, hospital administrator, and nursing officer‐in‐charge, with the medical superintendent as the chair. The health management team (HMT) was also answerable to the CHMT. The CHMT comprised the heads of subcounty program officers and departmental heads, with the county director of health as the chair. The county director of health was answerable to (*a*) the chief officer of health (also referred to as accounting officer) on financial issues and (*b*) the county executive member for health (CEC‐health) on technical matters such as planning. The chief officer of health was answerable to the CEC‐health and also accountable to the county treasury and the county assembly on financial issues. The CEC‐health was answerable to the county executive committee (CEC) that was chaired by the county governor. The CEC was then accountable to the county assembly.

#### 3.1.2 Formal planning and budgeting processes (on paper)

The CDOH was required to prepare an Annual Work Plan (AWP) that identifies activities to be implemented in the coming fiscal year and develop quarterly budgets that allocate resources against these activities. In line with the Public Finance Management Act (2012), the CDOH was required to use a program‐based budgeting method.

The planning and budgeting processes are expected to start in September with the AWP performance review and identification of subsequent years' priorities. In the same month, the county treasury releases the County Budget Review and Outlook Paper. The County Budget Review and Outlook Paper outlines the previous financial year's budgetary performance and presents projected county resource basket made of locally generated revenue and allocations from the national government, including the indicative allocations to all departments in the county. The priorities identified earlier on should inform the CDOH resource bidding process in December where each department undertakes lobbying for an increase or maintenance of budgetary allocation. This is followed by the release of the county fiscal strategy paper in February. The county fiscal strategy paper outlines broad county fiscal strategic priority goals that county departments should align their budgets and AWPs to. By end of April, the CDOH should have aligned their AWP with budget. This is followed by consolidation of the AWP and submission of the CDOH budget to the treasury where it is consolidated with other departmental budgets to form the county budget, which is taken for public participation. After public participation, the budget goes back to the departments for alignment with public views. The budget is then taken to the CEC for approval. After the CEC approval, the budget is taken to the county assembly budget committee who scrutinizes proposed allocations and expenditure to all departments. The committee invites public participation in this process. This is followed by submission to the full county assembly for final approval by the end of June.

#### 3.1.3 County planning and budgeting processes in practice

##### Misalignment between the AWPs and the budgets

While the AWP process and budgeting processes should be aligned, in practice, they were not aligned in both case study counties. The AWPs were prepared at least one quarter late, which implies that the budgets that were prepared prior to the AWP were not informed by the priorities laid out in the AWP.

The AWP starts in January of next year when actually the budget process starts in September of this year. So you are like three months behind so by the time they sit, all teams with the vastness of the county, by April we have not managed to meet. So, this is where it goes a little bit wrong … yes. Otherwise, now the AWP should be tied with the budget in the budget process, yeah. Senior manager, county A

In both counties, reasons for misalignment included the following: (*a*) the fact that the 2 processes are initiated and driven by different set of actors—the chief officer led the budgeting process and was accountable on financial matters to the county assembly and county treasury who strictly followed the budget timelines irrespective of the progress in the planning process, meanwhile the planning process was led by the county director who was not accountable to either the county treasury or the county assembly; (*b*) the perception by some senior managers that the budget was a legal requirement (enshrined in the Public Finance Management Act) while the AWP was not also thought to contribute to the misalignment; and (*c*) the fact that AWP timeline was “donor driven” thus dependent on the timelines of donors that supported the process.

While the budget process is spear headed by the Chief Officer and Principal Accountant, the Annual Work Plan is spear headed by County Director, CHMT and the Sub‐county teams, so it is a bit of a gap yes. Senior manager, county B

The Annual Work Plan is not a legal requirement. So sometimes you may find that people develop an Annual Work Plan in June but they don't complete it maybe until September. So you've already started implementing that year's budget but the Annual Work Plan may not be complete. Senior manager, county B

The AWP calender has not been formally integrated with the financial/budgeting calender. So in january when you receive a circular concerning the AWP, that is when you realize that you have developed a budget but not yet developed the AWP. Senior manager, county A

#### 3.1.4 Decision‐making criteria for planning and budgeting

Several formal and informal criteria were used in the planning and budgeting processes. Formal criteria are objective criteria that were used explicitly to set priorities. Informal criteria refer to subjective considerations that influenced decision making.

##### Formal criteria

In both counties, the dominant formal criteria used were essential services. These were services that were thought to be crucial in the delivery of healthcare. It was reported that the prominence of this criteria was driven by resource scarcity.

You look at what must be there, you cannot provide health care without drugs so that becomes one, you cannot run services without health workers. The “must be there” become top in the list then you look at those other issues Senior manager, county A

There are some things we cannot do without like electricity. You have to pay for electricity first and if you pay for electricity and then when the patients are there, patient food. You cannot do away with that one. Then drugs all the types of drugs the non‐pharms and the pharmaceuticals. That is the list of priorities Middle‐level manager, county B

Alignment with policy guidelines was an important consideration used by managers in decision making. For example, it was stipulated in the Public Finance Management Act, 2012, that 30% of the county's government budget has to be allocated to the development expenditure and 70% to the recurrent expenditure.

So if you've been allocated 2.5 billion, they [comptroller of budgets] just calculate what is 30% of that because that's the law and they tell you, your budget for development is this much, for recurrent is this much because it's law. Senior manager, county A

The Constitution and the Public Finance Management Act requires that at least 30% of county funds should be allocated to development. But because our county was a bit behind in infrastructure the county leadership decided to put more money in development. So instead of the 30%‐70% it's 52% development, 48% recurrent. Senior manager, county B

Another formal criterion that informed decision making was the performance of healthcare indicators. Priority was given to service areas whose health indicators performed poorly.

Our priority activities are also based on our indicators. Yes, we prioritize the places where we feel our indicators are not doing so well so we start by there. Middle‐level manager, county A

When we do the Annual Work Plan, we look at some indicators, how they are performing, and where we are not performing well based on the last year's performance we pick some items which we prioritize. Senior manager, county B

Decisions were also informed by previous AWPs and budgets.

The process of fund allocation basically was informed by the previous allocations. It was not really informed by what the work plan wants to execute. Senior manager, county B

We just sit and use the old plans so that they can inform us of the activities and we do this one because it is needed fast. So, it didn't follow the correct procedure. Middle‐level manager, county A

Other formal criteria used included accessibility, urgency of situation, revenue‐generating capacity, and the impact of activities or projects.

##### Informal criteria

In both counties, political interests had a major influence in the allocative decisions. However, in county A, political influence was more perverse, which led to perceptions of unfairness. This was linked to the fact that funds were centralized in county A hence easily accessible by the county leaders. In county A, all the funds for the 10 departments (including the Department of Health) were deposited in one bank account controlled by the county treasury while in county B, each department including hospitals owned and controlled their own bank accounts.

We had planned to procure 35 motorbikes for the rural public health officers to go to the field but the budget was cut down because the governor came and said that he had promised people that he will do a different activity but did not have the funds to do it. So, he said he will use the funds allocated to motorbikes to do what he promised people he would do. Senior manager, county A

The members of the county assembly have a very big influence on what project needs to be done. Because they will look at how much was allocated in the ward. And whether that project is a priority or not in the health department, but to them is a priority because they would want their ward to have something done. Senior manager, county B

Donors and health partners' priorities also influenced decisions. Their interests were factored in because of their ability to fund and support the activities carried out by the CDOH. However, sometimes their interests were not a priority for the department but since they have the funding, they were prioritized.

The role of partners in the planning is that they also have interests. They're also stakeholders because they implement health activities in the region. They may have an activity which they want to implement, but it is not a priority to us. So, they'll ask that we include it because they will support it financially. Middle‐level manager, county A

### 3.2 Evaluation of the planning and budgeting processes

#### 3.2.1 The use of consequentialist principles

##### Efficiency

Respondents from both counties appreciated the need to incorporate efficiency considerations in decision making. However, efficiency considerations were not incorporated for several reasons that included, among others, limited technical capacity, and a perception that government/public sector operations were not compatible with the use of efficiency criteria in decision making. It was also reported that political interests hindered the use of efficiency criteria.

When delivering government services, you don't need to consider costs or cost‐effectiveness because if you did, you would have to stop delivering some services. We don't consider cost‐effectiveness, but we consider the service delivery. Middle‐level manager, county A

These are just service delivery officers: nurses, doctors, clinical officer, public health officers during planning. The lack of technical skills in costing and cost‐effectiveness is a factor. They need some support on these skills and to always be reminded to factor in cost effectiveness or equity and fairness. Health partner county A

##### Equity

In both counties, equity was not incorporated in decision making. This was attributed to, among others, the lack of involvement of all the relevant stakeholders, the historical budgetary allocations, and most prominently, political interests.

There is a location in our sub‐county that is highly populated. This also has people that are very vocal politically, and very enlightened. This location tends to get more resources compared to others. For example it has more investment in health facilities. There's a facility here, the next facility is just next door. Middle‐level manager, county B

We know where the priority is as technocrats, as professionals, but an MCA [member of county assembly] will say I want a dispensary here and if you look at the distances, and the workload for that facility, it does not call for a new facility in that location. But they [MCAs] will say I want a dispensary here. So who are you to refuse? They [MCAs] are the guys who say if I won't get this dispensary here, then I'll not pass this motion[in the county assembly]. County manager, county A

##### Stakeholder satisfaction

Study respondents in both counties reported greater satisfaction with the planning process compared with the budgeting process. This was because the planning process was generally more inclusive than the budgeting process as the 2 processes were driven by different actors with different reporting and accountability relationships.

The AWP process is okay … but the budgeting process needs a lot to be done to become more inclusive … there should be a lot of involvement with us and the lower cadres especially the hospitals. It should not be just the county; they can have the vision, they should share with us so that we can also give our inputs before they make the final. Middle‐level manager, county A

We are not really satisfied (with the budgeting process) because had it been more inclusive and given sub‐counties and facilities more say, I believe things could have been better. Middle‐level manager, county B

##### Stakeholder understanding (awareness)

In both counties, stakeholder understanding was dependent on the extent of involvement in the planning and budgeting processes. For instance, in county A, since the managers at the dispensary level were not involved in both the planning and budgeting they had low understanding of the processes. Also, senior managers were more aware of the budgeting process compared with middle‐level managers. In county B, all the managers had a good understanding of the planning process. However, in the budgeting process, the middle‐level managers and the frontline managers had a low understanding.

At least I've told you the only two areas that we are involved fully is the Annual Work Plan and the Annual Development Plans …. It goes grey after that. It's a grey area, so unless you go looking for that information. The current situation doesn't really put you in an area where you can say you have understood the budgeting process. Middle‐level manager, county B

##### Shifted priorities (reallocation of resources)

In both counties, the recurrent section of the planning and budgeting processes, which consumed around 70% of the resources, did not result in shifted priorities. This was because, as indicated earlier, these processes were guided by historical allocations.

I would say that about 60‐70% of the budget is a bit of cut and paste, with the latitude for new and fresh things is just about 30%. Middle‐level manager, county B

There is nothing you can do to change recurrent expenditure. If it is salaries, its salaries throughout. If it is fuel it is fuel throughout. If it is commodities and supply it will be like that throughout. The only thing that maybe may not be appearing every other time is capital expenditure like purchases of vehicles, and infrastructure development. Senior manager, county A

##### Implementation of decisions

In both counties, planning and budgeting decisions were not fully implemented. This was blamed on the lack of funds. In county A, the managers felt that the unavailability of funds was worsened by the fact that CDOH service delivery units did not have decision space over resources. The low implementation of decisions had led to the perception of the planning process as having no importance.

Devolution meant decentralization of resources. In this county, we have not further decentralized services to the departments. Things are still run by the county chief officer of finance. Here (CDOH) we just sign papers and then they are taken to the county level who operate an account and can issue a cheque to support implementation. That's why sometimes decisions are not fully implemented. Senior manager, county A

Work plans are done well … very good documents, very good targets, objectives and activities but you find that they're not implemented because of the funding gap. Senior manager, county B

#### 3.2.2 Compliance with proceduralist conditions

##### Stakeholder engagement

In both counties, stakeholder engagement varied between the 2 processes, with the planning process being more inclusive than the budgeting process. It was also evident that the planning process was more inclusive in county B than in county A.

In the planning process in county A, health non–governmental organization, HMTs, SCHMTs, and the CHMT were involved. However, the public and the PHC‐FMTs were not involved. In the budgeting process, there were contrasting views on those who were involved. The senior managers reported to have involved the public, HMTs, and SCHMTs while these managers felt that they were not actually involved in the budgeting process. These contrasting responses were because the hospital and subcounty managers were invited but did not feel empowered in the budgeting process. The PHC‐FMTs were again completely excluded in the budgeting process. The reason for the noninvolvement of the frontline managers was attributed to lack of funds and time to involve them.

If I'm called today by the county office and I'm told that next week we need your annual work plan or your budget, I will not have time to involve them [sub‐county managers and frontline managers] because it is urgent. So I will just sit and take my mind to those areas so that I can plan for them. Middle‐level manager, county A

In the planning process in county B, health non–governmental organization, PHC‐FMTs, HMTs, the SCHMTs, and the CHMTs were involved. However, the public were not directly involved in making the AWPs. It was assumed that the hospital and facility committees would have contributed to the AWPs at the facility level. In the budgeting process, only the CHMT and the public were involved.

With the budgeting I think we can improve more in involving the facility in‐charges, involving medical superintendents and also the MOHs, sub‐county MOHs. I think we can involve those people more. Middle‐level manager, county B

##### Stakeholder empowerment

Stakeholder empowerment refers to the ability of a stakeholder to meaningfully contribute to the planning and budgeting processes. In county A, the PHC‐FMTs were not empowered as they were not given any opportunity to voice their concerns. The public were also not empowered in the planning process as they were not involved in it. Although the hospital and subcounty HMT were invited to the budgeting process, they felt that their involvement at that later stage was just to “rubber‐stamp” the county managers' decisions.

I remember last year we were told we were being invited to a budgeting process, but they had already allocated funds so, it's not really budgeting. We should budget together. We should all agree on priorities and allocate available funds. Middle‐level manager, county A

In the planning process in county B, only the public were not empowered because they were not involved. However, in the budgeting process, the SCHMTs, hospital HMTs, and PHC‐FMTs were not empowered as they were not involved in the deliberations of budgetary allocations within the department.

When it comes to the budgeting process we are not involved. So, it's not even a matter of being taken seriously or not. We are just not invited there Middle‐level manager, county B

##### Transparency

In both counties, stakeholders' perceptions on transparency were dependent on whether they were involved in the process. For this reason, they felt that the planning process was transparent while the budgeting was not. They also cited the lack of communication and feedback including rationales for decisions made as reasons for the low transparency of the budgeting process.

I've never seen a county budget. You will just hear in the local meetings that a certan dispensary has been allocated four million shillings but you'll never see that four million shillings. So I do not know who does those county budgets. They [county officials] know better. Frontline manager, county A

Communication is the biggest gap. When it comes to the transparency, with the AWP there is a lot of transparency. But the budget can be done and then when it's done, it's not communicated. Middle‐level manager, county B

##### Use of quality information

In both counties, information/data were scarcely used in decision making. This is because decisions were significantly driven by historical allocations. Other reasons reported were inaccurate and low‐quality data and political influence.

The major factor that was used is previous budgets and experience. We have decided going forward we, we want to be scientific. Senior manager, county A

##### Revisions

In both counties, there were no formal mechanisms to appeal the decisions made; therefore, the approved decisions cannot be revised. So any emerging pressing priorities in the course of the implementation period can only be incorporated through a supplementary budget according to the provisions and timelines stipulated in the law.

Whatever activities you're planning in the AWP that is what will go unless an emergency has occurred. That's when we're supposed to put a supplementary budget. But we're supposed to work as per our AWP. Frontline manager, county A

We have never changed our work plan, I have never seen that. No, never seen that. Senior manager, county B

##### Community values

In both counties, there was little incorporation of community values during the planning and budgeting processes. The only mechanism currently incorporating the needs of the community is through public participation in the budget process. The budget lacks the details of activities that will be carried out, which are usually in the AWP. This makes the public participation a forum to mostly discuss the development activities, which consume only 30% of the resources. The managers in both counties felt that the major challenge in incorporating community values was the lack of technical capacity and awareness by the public to make informed decisions.

You could go to a public participation and they tell you, “We want our own ambulance in our village. We want a maternity wing in our own village. We want a new facility in our village.” But if you look at where the health facility is, it's less than a kilometre. But the community has said they want their own facility. Senior level manager, county B

## 4 DISCUSSION

County health departments play a critical role in the Kenyan health system because they not only control the biggest proportion of healthcare resources but are also responsible for healthcare service delivery. To effectively deliver on their role, it is imperative that they have strong and systematic priority setting processes. We make several observations from this study. First, whereas the planning and budgeting processes of county health departments are expected to be aligned, in practice, these processes are not aligned in the 2 case study counties. Lack of alignment weakens the effectiveness of planning processes because it is likely that AWPs which reflect county priorities, are not backed by budgets and hence are unlikely to be implemented. An interesting observation is that some senior health managers did not feel compelled to align the two processes because of the perception that the planning process was not backed by any legal framework, while the budgeting process was enshrined in the public finance management law. Misalignment between the planning and budgeting processes is not a new occurrence within the health sector and is well documented in several studies in LMICs.^6^, ^19^, ^20^, ^21^ For example, a study done in 9 African countries to examine the implementation of the Medium Term Expenditure Framework as a solution to the misalignment found that context‐specific factors and political circumstances were a barrier to the intended alignment of the processes.^21^

Second, county health priorities appear to be significantly influenced by informal considerations, predominantly political and donor interests. This is an important consideration in the context of political decentralization in Kenya. Kenya's devolution has resulted in the establishment of a local political structure that appears to have direct influence on healthcare priority setting decisions. Without adequate safeguards, the influence of political interests could result in inequitable allocations by, for example, prioritizing subcounty regions with the most vocal or powerful political representations, regions that have demonstrated political support for current county political leaders, or regions that vote rich and therefore present a political opportunity for political leaders. The influence of politics and donors in regional level decision making is consistent with findings in other LMICs.^7^, ^22^, ^23^, ^24^ For instance, in Mbarali district in Tanzania, Maluka et al^22^ observed that many health facilities were constructed towards the election period irrespective of the district's priorities. The use of informal decision‐making considerations in decision making could lead to perception of unfairness and compromise the health system goals of equity and efficiency.

Third, the priority setting practices of the CDOH did not comply with the consequential conditions of the study's evaluative framework. For instance, there was no systematic attempt to incorporate efficiency and equity in decision making. The failure to use systematic approaches to guide priority setting in LMICs has been reported in a recent review of empirical literature on priority setting.^7^ Barriers to the incorporation of efficiency included limited technical capacity and a perception that efficiency considerations were not compatible with public sector decision making. These findings are different to what was found in Tanzania where the lack of quality data in the district was attributed to be a main cause of failure to consider efficiency.^22^ Political interference was regarded as a significant barrier to the incorporation of equity in decision making. Similar findings on politically induced inequities have been reported in both high‐income countries (HICs) and LMICs. For instance, in Tanzania, there was a politically motivated shift in priority from malaria to HIV/AIDs irrespective of the fact that the former had higher morbidity and mortality rates.^22^ Dionne et al also reported that strong political groups in Canada could advocate for their strategies leading to low priorities being ranked highly in the region.^25^ The findings also reveal that consequentialist outcomes of stakeholder satisfaction and understanding were not met in the budgeting process of both counties and the planning process in county A. This was linked to the fact that the 2 processes were not considered inclusive and transparent. The planning and budgeting processes also did not achieve shifted priorities. Empirical evidence suggests that the use of priority setting tools in decision making leads to proposal for reallocation of resources.^7^, ^26^, ^27^ The fact that tools were not used in the planning and budgeting may have contributed to the inability to identify areas for reallocation of resources. This study also reveals that the implementation of planning and budgeting decisions was unsatisfactory. The failure to fully implement decisions was mainly attributed to the scarcity of resources. However, it was also evident that the reduced financial autonomy in county A made it harder for them to implement decisions. Other studies done in LMICs and HICs have also demonstrated the influence of contextual factors on priority setting processes.^6^, ^26^, ^27^, ^28^ For instance, in Plymouth Primary Care Trusts in England, it was difficult to implement priority setting decisions that represented major cultural shifts and those that had major financial implications.^28^

Fourth, this study shows that county‐level healthcare priority setting practices do not meet the procedural conditions that guarantee deliberative democracy. This is because there was a lack of genuine commitment to engage stakeholders effectively through deliberations and empowerment. This lack of deliberations could be interpreted as a manifestation of power and a means by seniors to avoid scrutiny of their decisions by those below them. Similar findings have been reported in a study done in Canada where the senior managers found a way to bypass staff‐determined priorities by exempting executive‐determined priorities from scrutiny.^25^ This study also showed that involvement without empowerment is futile. It is important that stakeholders feel that their contributions are taken seriously. Both the planning and budgeting processes cannot be deemed as being transparent. This is because the communication strategies deployed were not reaching all the relevant stakeholders and that there was no communication of rationales for decisions. This finding is in tandem with several studies in both HICS and LMICs that have found that even when there is some communication, the sharing of rationales for decisions is not a tradition that leaders practice.^29^, ^30^, ^31^, ^32^, ^33^ Consistent with findings in other setting, there was no formal mechanism to appeal and revise the approved decisions.^31^, ^32^, ^33^, ^34^ It is well documented that through revisions, decision makers are able to improve the quality of decisions as it provides an opportunity to include emerging issues and to correct errors.^35^ There are limited mechanisms of incorporating community values in the planning and budgeting processes. This finding is consistent with studies done in both HICs and LMICs.^15^, ^22^, ^36^ According to communitarian claims, it is not enough to just subject the community to the decisions made, they should be involved to the extent of determining how resources are allocated.^37^ Public participation is the only mechanism currently used to incorporate public views. The main challenges encountered in public participation were lack of capacity and awareness. These findings mirror those found in Zambia and Tanzania where lack of interest and low literacy levels led to low public meeting attendance and lack of interest in expressing their concerns during the meetings.^24^, ^31^ Historical budgeting, political interference, and poor quality of data hindered the use of data for decision making.

Based on the findings of this study, we make several recommendations. First, the planning and budgeting processes should be harmonized. This can be achieved through (1) strict adherence to planning and budgeting activities and timelines as outlined in the policy and legal documents. (2) There is need for actors involved in driving the 2 processes to have similar and uniform reporting and accountability relationships so that they act in unison. Second, county governments should develop and implement a systematic priority setting process with explicit resource allocation criteria. Such a systematic process could incorporate efficiency and equity considerations in decision making. Third, there is need to improve the inclusivity of the planning and budgeting processes by including the public and frontline managers in the planning process and the frontline managers and middle‐level managers in the budgeting process. This will also improve stakeholder understanding and satisfaction. Fourth, there is need to empower all relevant stakeholders involved in the planning and budgeting processes. Empowerment should start with creating awareness on the importance of the AWPs and their role in the budgeting process, including the public, frontline managers, and middle‐level managers in the planning and budgeting processes, and communication of the AWPs and budgets including rationales for decisions. Fifth, there is need to strengthen and improve the existing channels of incorporating community values in the decision making. This can be achieved by involving hospital and facility health committees in the AWP process, including the AWPs in public participation, and using more channels of public participation of both AWPs and budgets such as through radio, television, and online debates.

### 4.1 Limitations

This study has a number of limitations. First, the community, politicians, and representatives from the Department of Treasury were not interviewed. These would have enriched the findings due to the crucial role they play in the process. Second, nonparticipant observation of the planning and budgeting processes would have strengthened the evidence by facilitating further methodological triangulation with the interviews and document review and increased the internal validity of the study. Third, this study focused on 2 counties out of 47 in the country, hence the inability to generalize the findings to other counties in Kenya. However, this study provides rich insights on how subnational governments can improve the planning and budgeting processes. These insights are transferable to similar settings.

## 5 CONCLUSION

Under the devolved system of government, county governments play a critical role in the delivery of healthcare services and control a significant proportion of healthcare resources. The performance of the Kenyan healthcare system is therefore dependent on how well the resources are managed by county health departments. Strengthening the priority setting process, specifically the planning and budgeting processes of county health departments, is therefore critical. This study has evaluated the planning and budgeting processes in 2 counties and highlighted both consequential and procedural aspects of the processes that need strengthening. Strengthening these aspects of the CDOH priority setting processes will strengthen the performance of the Kenyan health system by improving efficiency, equity, and responsiveness of service delivery.

## CONFLICT OF INTEREST

The authors declare that they have no conflicts of interest.

## AUTHOR CONTRIBUTION

D.W. did data collection, data curation, formal analysis, methodology, validation, writing–original draft preparation, and writing–review and editing. B.T. did conceptualization, formal analysis, funding acquisition, methodology, validation, and writing–review and editing. E.K. did methodology. E.B. did conceptualization, formal analysis, methodology, validation, writing–review and editing
